# Dynamic antimicrobial resistance and phylogenomic structure of *Salmonella* Typhimurium from 2007 to 2019 in Shanghai, China

**DOI:** 10.1128/spectrum.00262-24

**Published:** 2024-06-21

**Authors:** Zengfeng Zhang, Mengjun Hu, Xuebin Xu, Chao Lv, Chunlei Shi

**Affiliations:** 1 Department of Food Science & Technology, School of Agriculture and Biology, State Key Lab of Microbial Metabolism, Shanghai Jiao Tong University, Shanghai, China; 2 Laboratory of Microbiology, Shanghai Municipal Center for Disease Control and Prevention, Shanghai, China; 3 Department of Animal Health and Food Safety, School of Global Health, Chinese Center for Tropical Diseases Research, Shanghai Jiao Tong University School of Medicine, Shanghai, China; University of Maryland Eastern Shore, Princess Anne, Maryland, USA

**Keywords:** *Salmonella *Typhimurium, antimicrobial resistance, phylogenomic analysis, IncI (Gamma) plasmids

## Abstract

*Salmonella enterica* serovar Typhimurium is an important foodborne pathogen associated with human salmonellosis worldwide. A retrospective screening was performed to elucidate the prevalence, antimicrobial resistance, and phylogenomic characterization of this pathogen in Shanghai, China. *S*. Typhimurium isolates were selected from 2,211 serotyped *Salmonella* isolates collected during 2007–2019. Two hundred and seventy-seven *S*. Typhimurium isolates were detected in 15 of 16 districts in Shanghai. It was noted that 214 (77.3%) isolates were multi-drug resistant and 32 (11.6%) isolates were resistant to ciprofloxacin and 5 (1.8%) isolates were further resistant to ceftriaxone. Poisson generalized linear mixed model results showed that the multi-drug resistance (MDR) in 2017 and 2018 was significantly higher than that in 2010 (*P*＜0.05), highlighting an increase in the risk of MDR. Phylogenetic results showed that a global data set of 401 sequenced *S*. Typhimurium isolates was classified into four clones (ST36, ST313, ST19, and ST34), which appeared in international clonal dissemination. The ST34 isolates from China fell into two clades, ST34C1 and ST34C2, the latter of which might originate from Shanghai, and then expanded nationally, accompanied by extended-spectrum β-lactamase gene *bla*_CTX-M-14_ and a mutation in quinolone resistance-determining region of the *gyr*A 87 site. Furthermore, *bla*_CTX-M-14_ linking to IS*Ecp1* upstream and ΔIS*903B* downstream was found in IncI (Gamma)-like plasmids, and the plasmid conjugation contributed to its horizontal transmission. To our knowledge, it is the first report of the epidemiological and phylogenetic characterization for *S*. Typhimurium including the emerged clade ST34C2 in Shanghai, warranting the necessity of surveillance for this high-risk pathogen.

**IMPORTANCE:**

Our study uncovered a widespread distribution of *Salmonella enterica* serovar Typhimurium isolates in Shanghai accompanied by the increase in antimicrobial resistance (AMR) especially MDR during a 10-year period, which filled in the gap about a long period of continuous monitoring of AMR in this pathogen in Shanghai. Meanwhile, we identified a new clade ST34C2 of *S*. Typhimurium with the acquisition of IncI (Gamma)-like plasmids mediated by extended-spectrum β-lactamase gene *bla*_CTX-M-14_ as well as *gyr*A 87 mutation, which had not been reported before. It was noted that IncI (Gamma)-like plasmids were reported in *S*. Typhimurium for the first time and conjugation could accelerate the spread of antimicrobial resistance gene *bla*_CTX-M-14_. These findings on the epidemic, antimicrobial resistance, and phylogenomic characterization for *S*. Typhimurium provide valuable insights into its potential risk to public health and also the basis for AMR prevention and control strategies in Shanghai in the future.

## INTRODUCTION

Salmonellosis is one of the most common foodborne diseases worldwide, which poses a serious threat to public health (1, 2). Among more than 2,600 serovars of *Salmonella*, *Salmonella enterica* serovar Typhimurium was a leading cause of human gastroenteritis worldwide (3). In China, *S*. Typhimurium was the most common serovar responsible for acute gastroenteritis among all ages of people, accounting for 69.1% (4). Furthermore, the outbreaks of *S*. Typhimurium were frequently reported to be caused by food contaminations. In 2018, a multistate foodborne outbreak of *S*. Typhimurium in the United States resulted in at least 265 persons with foodborne pathogen illness, and the source of the outbreak was prepackaged chicken salad (5). In the global sense, 369 cases (by 18 May 2022) of confirmed and probable *S*. Typhimurium linked to chocolate products from Belgium have been reported (6). Especially in Europe, the outbreak of multi-drug-resistant (MDR, resistance to at least three antimicrobial classes) monophasic *S*. Typhimurium associated with chocolate products had resulted in at least 150 reported cases (by 10 April 2022) in nine EU/EEA countries and the UK, and the cases were predominately under the age of 10 years (*n*  =  134; 89%) (7). Therefore, it is urgent to monitor the prevalence of *S*. Typhimurium to reduce its threat to food safety.

The increased AMR in bacteria has become a global public health concern. A previous study reported that in 2019, AMR directly resulted in more than 1.2 million deaths in 204 countries worldwide, which was higher than the number of deaths caused by HIV infection or malaria, while the number of indirect deaths was as high as 4.95 million (8). The AMR study commissioned by the UK government has estimated that approximately 10 million people will die annually by 2050 due to drug-resistant bacteria unless there is a global response to the problem of AMR (9). The overall mortality rate in patients infected with MDR pathogens in intensive care units was 13.1%, which was two to three times higher than those infected with sensitive strains (10). The high-level AMR, especially MDR, had been observed in *S*. Typhimurium isolates in a previous study from Henan, China, 91.1% of *S*. Typhimurium isolates was MDR, and 13 MDR isolates exhibited co-resistance to the cephalosporins and fluoroquinolones, which are the first-line antimicrobials for the treatment of pathogen infections (11). The classical MDR pattern of ACSSuT (ampicillin, chloramphenicol, streptomycin, sulphonamides, and tetracycline) ranged from 21.6% to 22.8% in *S*. Typhimurium isolates (12, 13). In addition to AMR, important antimicrobial resistance genes (ARG) such as *mcr*-1, *mcr*-3, and *bla*_CTX-M_ in *S*. Typhimurium isolates had expanded and spread worldwide (14–17). We are now facing a formidable and growing menace for the clinical treatment from the MDR *S*. Typhimurium isolates.

Currently, whole-genome sequencing has been widely applied to reveal the evolutionary dynamics of bacteria (18–20). In Africa, the most common *S. enterica* associated with invasive non-typhoidal salmonellosis is *S*. Typhimurium ST313 (19). AMR and genome degradation were found to contribute to the success of ST313 tree lineages (L1, L2, and L3) in the phylogenomic analysis (19). In Japan, the phylogenomic analysis showed that *S*. Typhimurium and its monophasic 4,[5],12:i:- could be divided into nine clades, and clade 9 (ST34) among food animals might be a part of the *S*. 4,[5],12:i:- clone in Europe (21). Currently, the evolutional characteristics of *S*. Typhimurium isolates are still unclear in China. It is important to elucidate the phylogenetic route of *S*. Typhimurium isolates to track their dissemination through phylogenomic analysis.

In this study, a large-scale retrospective screening was performed on 2,211 serotyped *Salmonella* isolates from Shanghai, China, to elucidate the regional prevalence and AMR characteristics of *S*. Typhimurium. Phylogenomic analysis was performed on *S*. Typhimurium isolates in this study together with those from other countries to gain insight into the population structure and evolutionary dynamics. In addition, plasmid sequence characteristics and plasmid transferability were further explored in this pathogen.

## RESULTS AND DISCUSSION

### The distribution and source analysis of *S*. Typhimurium isolates

**Fig 1:**
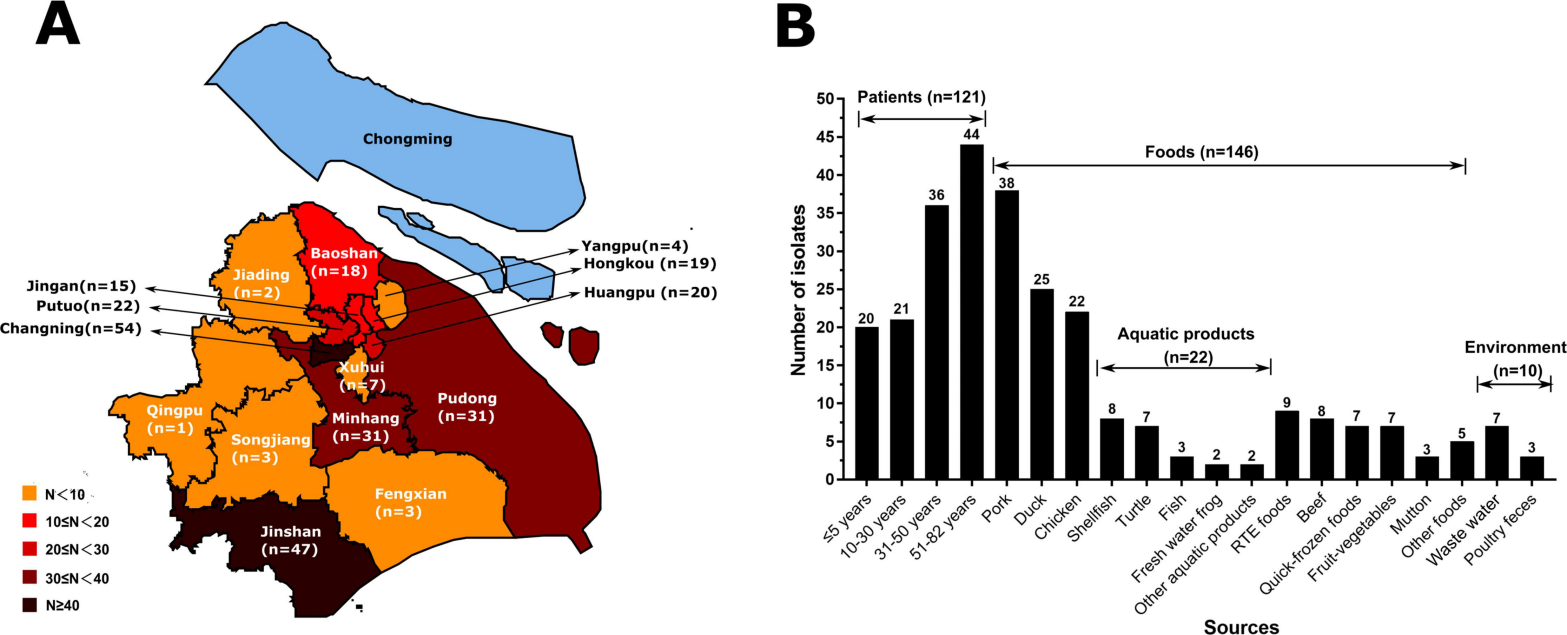
Prevalence of *S*. Typhimurium isolates in Shanghai, China. (**A**) The distribution of isolates in the districts of Shanghai city. The districts where *S*. Typhimurium isolates were identified are indicated in red, and the district where the isolate was not identified is indicated in blue-green. The number of isolates obtained from each district is shown. The initial map originated from DataV.GeoAtlas (http://datav.aliyun.com/portal/school/atlas/area_selector), followed by the use of Inkscape software to overlay different colored shades for representation. (**B**) The sample sources of isolates. The number of isolates obtained from different sources is shown.

A total of 277 (12.5%) *S*. Typhimurium isolates were obtained from 2,211 serotyped *Salmonella* isolates, which were disseminated in 15 of 16 districts in Shanghai (Baoshan, Fengxian, Hongkou, Huangpu, Jiading, Jinshan, Jingan, Minhang, Pudong, Putuo, Qingpu, Songjiang, Xuhui, Yangpu, and Changning) (Fig. 1A), suggesting their wide distribution in this city.

In this study, food samples (52.7%; 146/277) were the predominant sources of *S*. Typhimurium isolates (Fig. 1B). It was further demonstrated that food samples were mainly composed of pork, duck, chicken, and aquatic products. A total of 38 (26.0%) isolates were recovered from pork, accounting for the largest portion, and then duck (17.1%), chicken (15.1%), and aquatic products (15.1%) (Fig. 1B), which suggested that pork was the main vehicle of *S*. Typhimurium isolates. It was noted that *S*. Typhimurium isolates were identified in shellfish (*n* = 8) and turtle (*n* = 7) samples. In a previous study, the genetic relationship of *Salmonella* isolates from turtles and humans was close, which suggested a possible public health risk of *Salmonella* infections transmitted through turtles (22). In addition, *S*. Typhimurium isolates could be recovered from ready-to-eat foods, quick-frozen foods, and fruit-vegetables, which were rarely reported in previous studies. Besides, 121 (43.7%) *S*. Typhimurium isolates were recovered from patients whose ages ranged from 8 months after birth to 82 years old (Fig. 1B). Forty-four (36.4%) isolates were identified in patients from 51 to 82 years old, which were mostly the elderly with weakened immunity. Moreover, *S*. Typhimurium isolates could also be found in the environments, such as waste water and poultry feces.

### The AMR in *S*. Typhimurium

Among 277 *S*. Typhimurium isolates, the highest rate of resistance to sulfisoxazole (98.6%) was observed and then nalidixic acid (67.1%), ampicillin (61.0%), tetracycline (55.6%), trimethoprim-sulfamethoxazole (51.6%), streptomycin (47.3%), chloramphenicol (43.7%), gentamicin (42.2%), kanamycin (41.5%), and amikacin (0.4%) (Table 1). All 277 *S*. Typhimurium isolates were susceptible to colistin, meropenem, and imipenem. It was noted that 32 (11.6%) and 5 (1.8%) isolates were found to be resistant to ciprofloxacin and ceftriaxone, respectively, which are typically employed as first-line drugs for salmonellosis treatments upon other therapies have failed (23).

**TABLE 1:** Antimicrobial resistance of 277 *S*. Typhimurium isolates from patients, foods, and environment in Shanghai, China^*a*^

Antimicrobials	Resistant isolates (no./ratio %)			Total (*n* = 277)
	Patients (*n* = 121)	Foods (*n* = 146)	Environment (*n* = 10)	
*β*-Lactams				
Ampicillin	77/63.6^a^	85/58.2^a^	7/70.0	169/61.0
Ceftriaxone	4/3.3^a^	1/0.7^a^	0/0.0	5/1.8
Ceftiofur	4/3.3^a^	1/0.7^a^	0/0.0	5/1.8
Aminoglycosides				
Amikacin	1/0.8^a^	0/0.0^a^	0/0.0	1/0.4
Gentamicin	56/46.3^a^	60/41.1^a^	1/10.0	117/42.2
Streptomycin	57/47.1^a^	69/47.3^a^	5/50.0	131/47.3
Kanamycin	47/38.8^a^	65/44.5^a^	3/30.0	115/41.5
Quinolones				
Nalidixic acid	84/69.4^a^	96/65.8^a^	6/60.0	186/67.1
Ciprofloxacin	21/17.4^a^	10/6.8 ^b^	1/10.0	32/11.6
Tetracyclines				
Tetracycline	67/55.4^a^	81/55.5^a^	6/60.0	154/55.6
Sulphamethoxazole				
Sulfisoxazole	121/100.0^a^	142/97.3^a^	10/100.0	273/98.6
Folate pathway antagonists				
Trimethoprim-sulfamethoxazole	60/49.6^a^	78/53.4^a^	5/50.0	143/51.6
Phenicols				
Chloramphenicol	57/47.1^a^	61/41.8^a^	3/30.0	121/43.7
Lipopeptides				
Colistin	0/0.0	0/0.0	0/0.0	0/0.0
Carbapenems				
Meropenem	0/0.0	0/0.0	0/0.0	0/0.0
Imipenem	0/0.0	0/0.0	0/0.0	0/0.0
≥3 (MDR)	101/83.5^a^	107/73.3^a^	6/60.0	214/77.3
≥5	67/55.4^a^	78/53.4^a^	2/20.0	147/53.1
≥7	20/16.5^a^	12/8.2^a^	0/0.0	32/11.6

^*a*^ Different superscript lowercase letters on one line indicate a significant difference (*P* < 0.05).

**TABLE 2:** Antimicrobial resistance of 277 *S*. Typhimurium isolates from 2007 to 2019 in Shanghai, China^*a*^

Antimicrobials	Resistant isolates (no./ratio %)					
	2007–2011 (*n* = 73)	2012–2013 (*n* = 58)	2014–2015 (*n* = 55)	2016–2017 (*n* = 38)	2018–2019 (*n* = 53)	Total (*n* = 277)
*β*-Lactams						
Ampicillin	35/47.9^b^	30/51.7^ab^	43/78.2^a^	30/78.9^a^	31/58.5^ab^	169/61.0
Ceftriaxone	1/1.4^a^	0/0.0	2/3.6^a^	2/5.3^a^	0/0.0	5/1.8
Ceftiofur	1/1.4^a^	0/0.0	2/3.6^a^	2/5.3^a^	0/0.0	5/1.8
Aminoglycosides						
Amikacin	0/0.0	1/1.7	0/0.0	0/0.0	0/0.0	1/0.4
Gentamicin	19/26.0^b^	24/41.4^ab^	27/49.1^a^	19/50.0^a^	28/52.8^a^	117/42.2
Streptomycin	22/30.1^c^	24/41.4^bc^	31/56.4^ab^	24/63.2^a^	30/56.6^ab^	131/47.3
Kanamycin	20/27.4^c^	21/36.2^bc^	25/45.5^ab^	19/50.0^ab^	30/56.6^a^	115/41.5
Quinolones						
Nalidixic acid	41/56.2^b^	36/62.1^ab^	41/74.5^a^	29/76.3^a^	39/73.6^a^	186/67.1
Ciprofloxacin	1/1.4^c^	3/5.2^bc^	9/16.4^ab^	9/23.8^a^	9/17.0^ab^	32/11.6
Tetracyclines						
Tetracycline	33/45.2^bc^	25/43.1^c^	34/61.8^ab^	29/76.3^a^	33/62.3^ab^	154/55.6
Sulphamethoxazole						
Sulfisoxazole	73/100.0^a^	58/100.0^a^	55/100.0^a^	38/100.0^a^	49/92.5^a^	273/98.6
Folate pathway antagonists						
Trimethoprim-sulfamethoxazole	31/42.5^bc^	21/36.2^c^	34/61.8^a^	26/68.4^a^	31/58.5^ab^	143/51.6
Phenicols						
Chloramphenicol	17/23.3^b^	19/32.8^b^	31/56.4^a^	24/63.2^a^	30/56.6^a^	121/43.7
Lipopeptides						
Colistin	0/0.0	0/0.0	0/0.0	0/0.0	0/0.0	0/0.0
Carbapenems						
Meropenem	0/0.0	0/0.0	0/0.0	0/0.0	0/0.0	0/0.0
Imipenem	0/0.0	0/0.0	0/0.0	0`/0.0	0/0.0	0/0.0
≥3 (MDR)	48/65.8^b^	44/75.9^ab^	46/83.6^a^	32/84.2^a^	44/83.0^a^	214/77.3
≥5	30/41.1^c^	25/43.1^c^	29/52.7^bc^	28/73.7^a^	35/66.0^ab^	147/53.1
≥7	4/5.5^a^	7/12.1^a^	8/14.5^a^	5/13.2^a^	8/15.1^a^	32/11.6

^*a*^ Different superscript lowercase letters on one line indicate a significant difference (*P* < 0.05).

Ciprofloxacin resistance (17.4%) in isolates from patients was significantly higher (*P* < 0.05) than that (6.8%) from foods. There was no significant difference (*P*＞0.05) in resistance to sulfisoxazole, nalidixic acid, ampicillin, tetracycline, trimethoprim-sulfamethoxazole, streptomycin, chloramphenicol, gentamicin, and kanamycin. Five ceftriaxone-resistant isolates were recovered from patients (*n* = 4) and meat sample (*n* = 1). The ceftriaxone-resistant isolates in this study were found to carry the *bla*_CTX-M-14_ gene. The cephalosporin resistance was usually due to extended-spectrum β-lactamases (ESBLs) including the CTX-M group produced by *Salmonella* spp. (24–26). It was noted that SJTUF10405 and SJTUF11216 isolates from patients were concurrently resistant to both ciprofloxacin and ceftriaxone. Furthermore, these two isolates harbored a ACSSuT resistance pattern. The ACSSuT type had spread in *S*. Typhimurium isolates in the world (27). We further compared the AMR characteristics of isolates from different foods (Table S1). Ciprofloxacin resistance in isolates from aquatic products (13.6%) was significantly higher than that from meats (6.3%) (*P* < 0.05) (Table S1). A total of 214 (77.3%) *S*. Typhimurium isolates were identified to have MDR profiles (Table 1). Furthermore, 147 (53.1%) and 32 (11.6%) isolates were resistant to at least five and seven antimicrobial classes, respectively. The MDR rate (83.5%) was identified in isolates from patients in this study, which was consistent with that (84.6%) in *S*. Typhimurium isolates from patients with diarrhea in a previous study (12). The high-level MDR in *S*. Typhimurium isolates, especially ciprofloxacin and ceftriaxone co-resistance, would greatly increase the challenge of clinical treatments for patients.

It was noted that more than 50% of isolates from 2014 to 2019 was resistant to ampicillin, streptomycin, nalidixic acid, tetracycline, sulfisoxazole, trimethoprim-sulfamethoxazole, and chloramphenicol (Table 2), suggesting the severe threat to clinical treatment of infections. Furthermore, some AMR rates exhibited significant changes over the years. The gentamicin resistance rate significantly increased from 26.0% in 2007–2011 to 52.8% in 2018–2019 (Table 2). Similar results could be found in other aminoglycoside resistance such as kanamycin and streptomycin. The tetracycline resistance rate significantly increased from 45.2% in 2007–2011 to 76.3% in 2016–2017 (*P* < 0.05) and declined to 62.3% in 2018–2019. The chloramphenicol resistance rate significantly increased from 23.3% in 2007–2011 to 56.6% in 2018–2019 (*P* < 0.05). It was noted that the ciprofloxacin resistance rate significantly increased from 1.4% in 2007–2011 to 17.0% in 2018–2019 (*P* < 0.05). The nalidixic acid resistance rate significantly increased from 56.2% in 2007–2011 to 73.6% in 2018–2019 (*P* < 0.05). The sulfisoxazole resistance rate was maintained at a high level (92.5%-100.0%) over these 10 years. Meanwhile, both the ceftriaxone resistance rate and the ceftiofur resistance rate were maintained at a low level (1.4%–5.3%) over these 10 years. The MDR rate significantly increased from 65.8% in 2007–2011 to 83.0% in 2018–2019 (*P* < 0.05).

The Poisson generalized linear mixed models (GLMMs) were further used to test whether MDR differed among sampling sources, years, and locations (Table 3). The MDR in non-patient isolates was negatively correlated with that in patients [odds ratio (OR) = 0.957], and they were not significantly different (*P* > 0.05). MDRs in isolates from 2011 to 2016 were not significantly different with those in 2010 as reference (*P* > 0.05). However, MDRs in isolates in 2017 and 2018 were positively correlated with those in 2010 as reference (OR > 1), and a significant difference was observed (*P* < 0.05) (Table 3). In addition, MDR in eight locations (Hongkou, Huangpu, Jinshan, Jingan, Minhang, Pudong, and Putuo) showed no significant difference than that in Changning as reference (*P* > 0.05) (Table 3).

**TABLE 3:** Analysis of a Poisson generalized linear mixed model examining the likelihood of antibiogram length within the hosts, years, and locations

	No. isolates	Estimate	SE	*Z* score	*P* value
Sources					
Patients	84	Reference			
Non-patients	101	−0.044	0.069	−0.637	0.524
Years					
2010	30	Reference			
2011	19	−0.127	0.142	−0.891	0.373
2012	17	−0.125	0.147	−0.849	0.396
2013	22	0.083	0.128	0.652	0.514
2014	26	0.121	0.121	1.001	0.317
2015	12	0.280	0.145	1.931	0.053
2016	14	0.166	0.143	1.159	0.246
2017	15	0.277	0.135	2.049	0.040
2018	30	0.396	0.110	3.594	0.000
Locations					
Changning	36	Reference			
Hongkou	14	0.038	0.169	0.223	0.823
Huangpu	15	0.038	0.154	0.244	0.807
Jinshan	31	−0.103	0.132	−0.781	0.435
Jingan	11	0.006	0.197	0.032	0.974
Minhang	23	0.015	0.143	0.104	0.917
Pudong	21	−0.030	0.145	−0.206	0.837
Putuo	17	−0.005	0.167	−0.033	0.974

It appeared that the high-level MDR in *S*. Typhimurium was likely to be caused by the widespread use of antimicrobials. In 2013, the total amount of antimicrobials used in China was about 162,000 tons, approximately 160 times that of the United Kingdom, and 48% of which was used for human consumption and the rest was shared by animals (28). Furthermore, the production yield of fluoroquinolones (including ciprofloxacin) and β-lactams (including ceftriaxone) in China was estimated to be 27,300 and 34,100 tons, respectively (28). Governmental regulations limiting the use of antimicrobial agents have been issued in China to reduce the potential threat of MDR bacteria to the public health. A national plan against antimicrobial resistance 2016–2020 to strengthen national AMR prevention and control (https://www.gov.cn/xinwen/2016-08/25/content_5102348.htm) in China was issued. Some AMR rates had a declined trend in 2018–2019 compared with those in 2016–2017, such as ampicillin, ciprofloxacin, tetracycline, trimethoprim-sulfamethoxazole, and chloramphenicol, which supported the behavior of the national plan. Indeed, the Chinese government has issued a new national plan against antimicrobial resistance 2022–2025 to further control AMR (http://www.nhc.gov.cn/yzygj/s7659/202210/2875ad7e2b2e46a2a672240ed9ee750f.shtml). In addition, “One Health,” as a new concept and strategy for AMR control, is becoming a research hotspot globally (29). It emphasizes the holistic interconnectedness of animals, the environment, food, and humans and requires coordinated cooperation across fields and industries to address AMR. We believe that these strategies will contribute to the decrease of AMR in the future, which will greatly reduce the threat to public health.

### Phylogenomic analysis of *S*. Typhimurium isolates internationally

**Fig 2:**
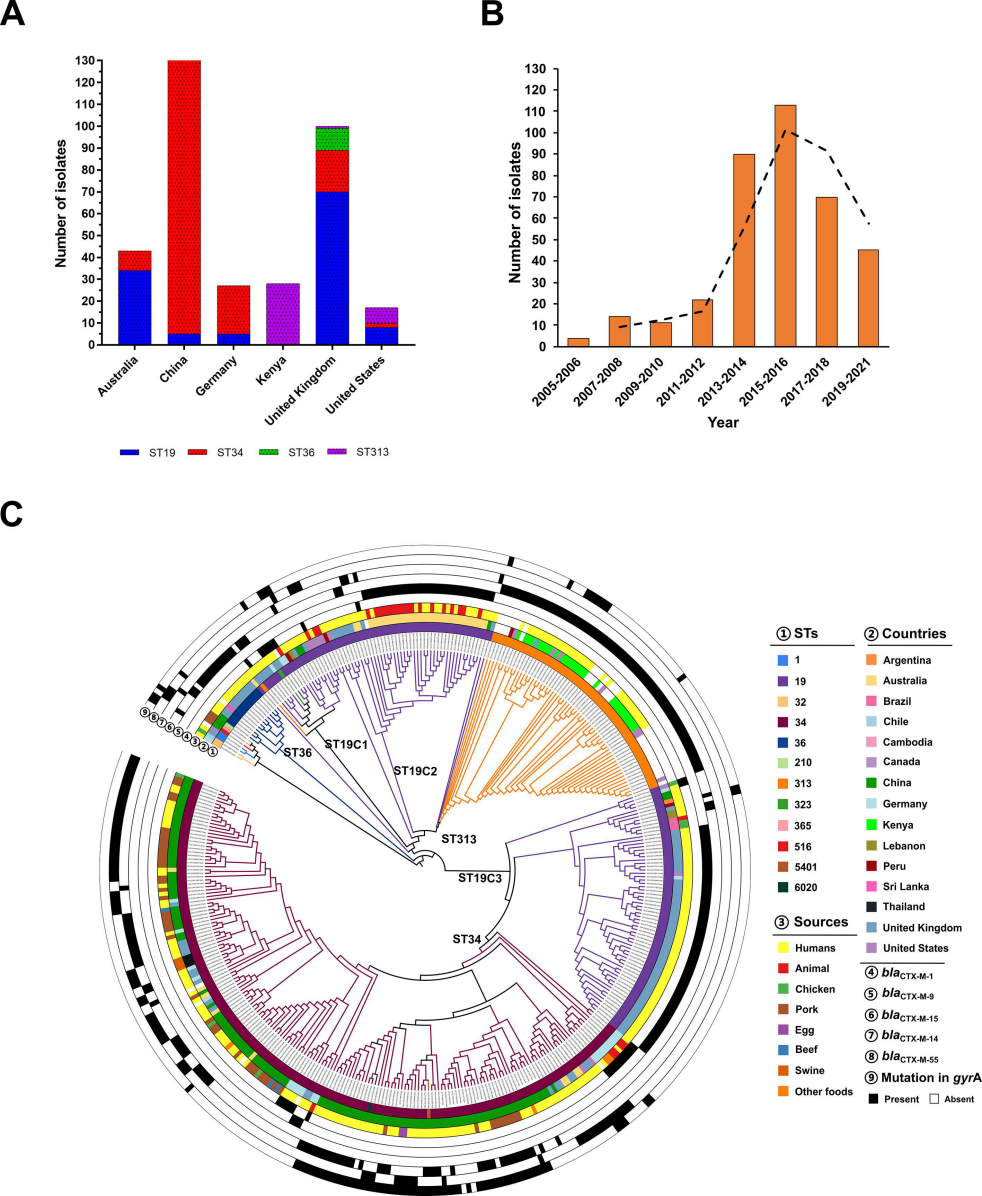
(**A**) ST distribution of *S*. Typhimurium isolates bearing *bla*_CTX-M_ in Australia, China, Germany, Kenya, the United Kingdom, and the United States. (**B**) The prevalence of *S*. Typhimurium isolates bearing *bla*_CTX-M_ from 2005 to 2021. (**C**) Phylogenomic analysis of *S*. Typhimurium isolates bearing *bla*_CTX-M_ from 15 countries. Rings ①–⑨ from inside to outside along the tree represent metadata including STs, countries, sources, and *bla*_CTX-M_ variants (as shown in the inset legend).

To determine the evolutionary dynamics of *S*. Typhimurium isolates carrying *bla*_CTX-M_, phylogenomic analysis was performed on 135 genomes from China and 266 genomes in the public databases from 14 other countries. Major types of ST34 (*n* = 183), ST19 (*n* = 133), ST313 (*n* = 66), and ST36 (*n* = 11) were identified in these 401 *S*. Typhimurium isolates. Furthermore, ST types varied in different countries. ST34 was the major type in China and Germany (Fig. 2A). ST19 was the major type in the United Kingdom and Australia. ST313 was the major type in Kenya. Importantly, there was a sharp increase after 2012 (Fig. 2B).

A total of 103,276 core single-nucleotide polymorphisms (SNPs) were extracted from these 401 *S*. Typhimurium genome sequences to construct a maximum likelihood tree (Fig. 2C). Phylogenomic results suggested that the clustering effect of *S*. Typhimurium isolates was indicated by their ST types. These *S*. Typhimurium isolates evolved into ST34, ST313, ST19, and ST36 clones (Fig. 2C ; Fig. S1). The ST36 clone was composed of 12 human isolates from the United Kingdom (*n* = 10), China (*n* = 1), and Sri Lanka (*n* = 1), most of which carried *bla*_CTX-M-15_. The ST19 clone was separated into ST19C1, ST19C2, and ST19C3 clades. Clade ST19C2 evolved from clade ST19C1, both of which showed a far phylogenetic relationship with clade ST19C3. Clade ST19C2 was mainly composed of isolates from Australia, most of which carried *bla*_CTX-M-9_. Clade ST19C3 was mainly composed of human isolates from the United Kingdom, most of which also carried *bla*_CTX-M-9_. The ST313 clone was composed of human isolates from Kenya and the United Kingdom, all of which carried *bla*_CTX-M-15_, suggesting that their genetic relationships were close. Furthermore, most isolates from China belonged to ST34 and fell into the ST34 clone (Fig. 2C). Besides the isolates from China, this ST34 clone also consisted of some isolates from the United Kingdom, Germany, Thailand, and Australia, showing their close genetic relationship. In the ST34 clone, it was observed that one historical, independent location jump event from China to Germany occurred in a 1998 and then a subsequent location jump back into China in 2005 (Fig. S2). Another location jump event across the United Kingdom and China occurred in 2004 (Fig. S2). The location jump events implied that the international spread of the *S*. Typhimurium ST34 clone occurred.

### Phylogenomic analysis of *S*. Typhimurium isolates in China

**Fig 3:**
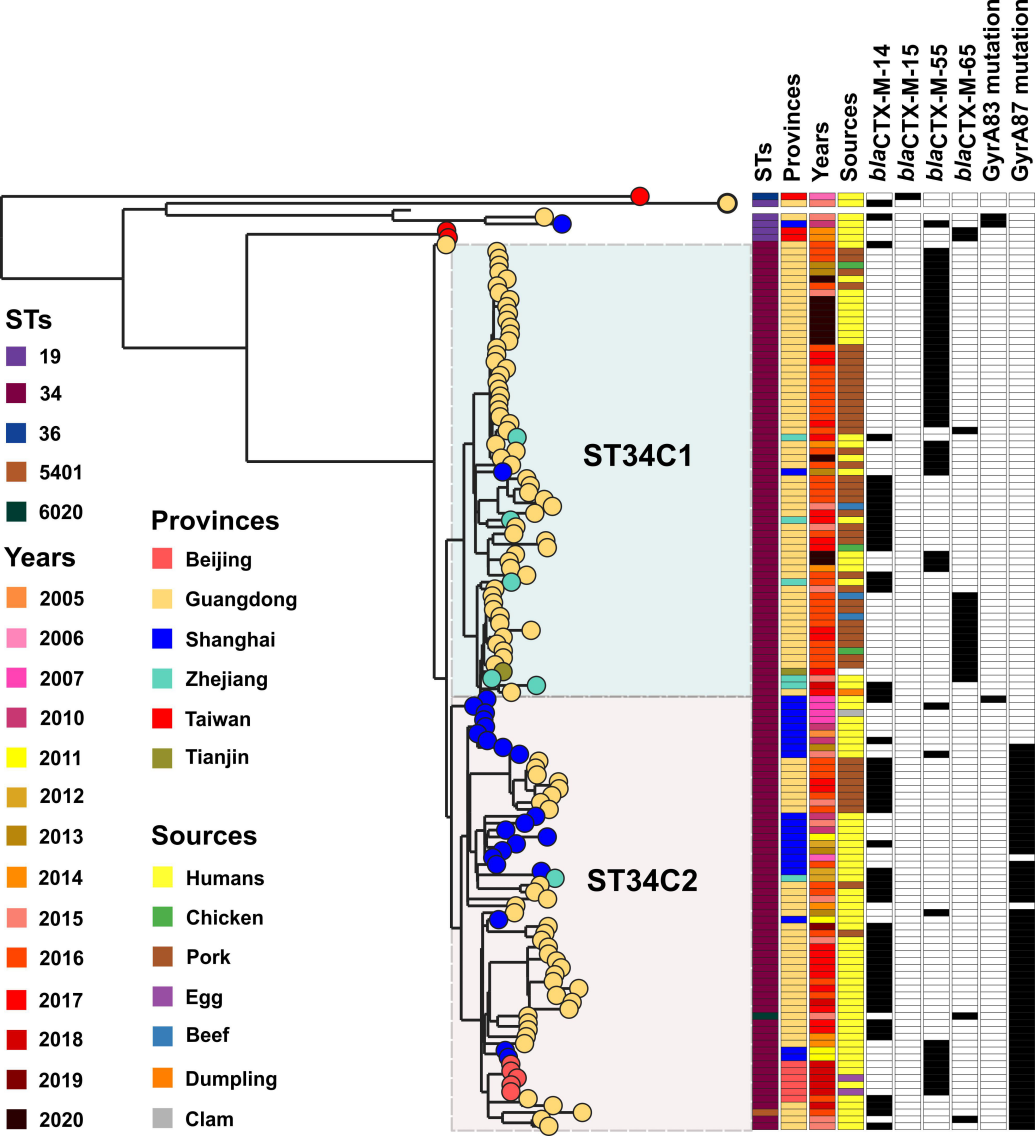
Phylogenetic tree of *S*. Typhimurium isolates bearing *bla*_CTX-M_ in China. Leaf nodes are colored by provinces (see the key). The colors of isolate tips represent metadata columns including STs, provinces, years, sources, *bla*_CTX-M_ variants (*bla*_CTX-M-14_, *bla*_CTX-M-15_, *bla*_CTX-M-55_, and *bla*_CTX-M-65_), and mutations in QRDR of GyrA (as shown in the inset legend). Light-green and pink shading shows clades ST34C1 and ST34C2, respectively.

To further understand the evolutional characteristics of *S*. Typhimurium isolates in China, a phylogenetic tree was re-constructed on 135 Chinese genomes including 16 genomes in this study (Fig. 3). It was found that ST34 isolates in China fell into clades ST34C1 and ST34C2, and isolates from Shanghai fell into clade ST34C2. It was noted that the base isolates of clade ST34C2 were from Shanghai and then evolved in Guangdong and Beijing. It was interesting that base isolates in Shanghai carried no mutation in *gyr*A, but the rest in ST34C2 obtained a mutation in the *gyr*A 87 site. Point mutations in the quinolone resistance-determining region (QRDR) of GyrA and ParC have been demonstrated to lead to decreased susceptibility to fluoroquinolones (30, 31). The mutation in *gyr*A might provide an evolutional power for ST34C2 isolates under external antimicrobial selective pressure.

ST34C1 was mainly composed of isolates from Guangdong and Zhejiang, and ST34C2 was mainly composed of isolates from Shanghai, Beijing, and Guangdong. The difference between ST34C1 and ST34C2 was that *bla*_CTX-M-55_ was the major type in the former but *bla*_CTX-M-14_ in the latter. Furthermore, another difference was that no mutation in *gyr*A occurred in the former. In addition, isolates from different sources (ST34C1: human, pork, chicken, and beef isolates; ST34C2: human and pork isolates) showed a close genetic relationship, suggesting that clone spread occurred.

### Plasmid sequence analysis of *S*. Typhimurium isolates carrying *bla*_CTX-M_

**Fig 4:**
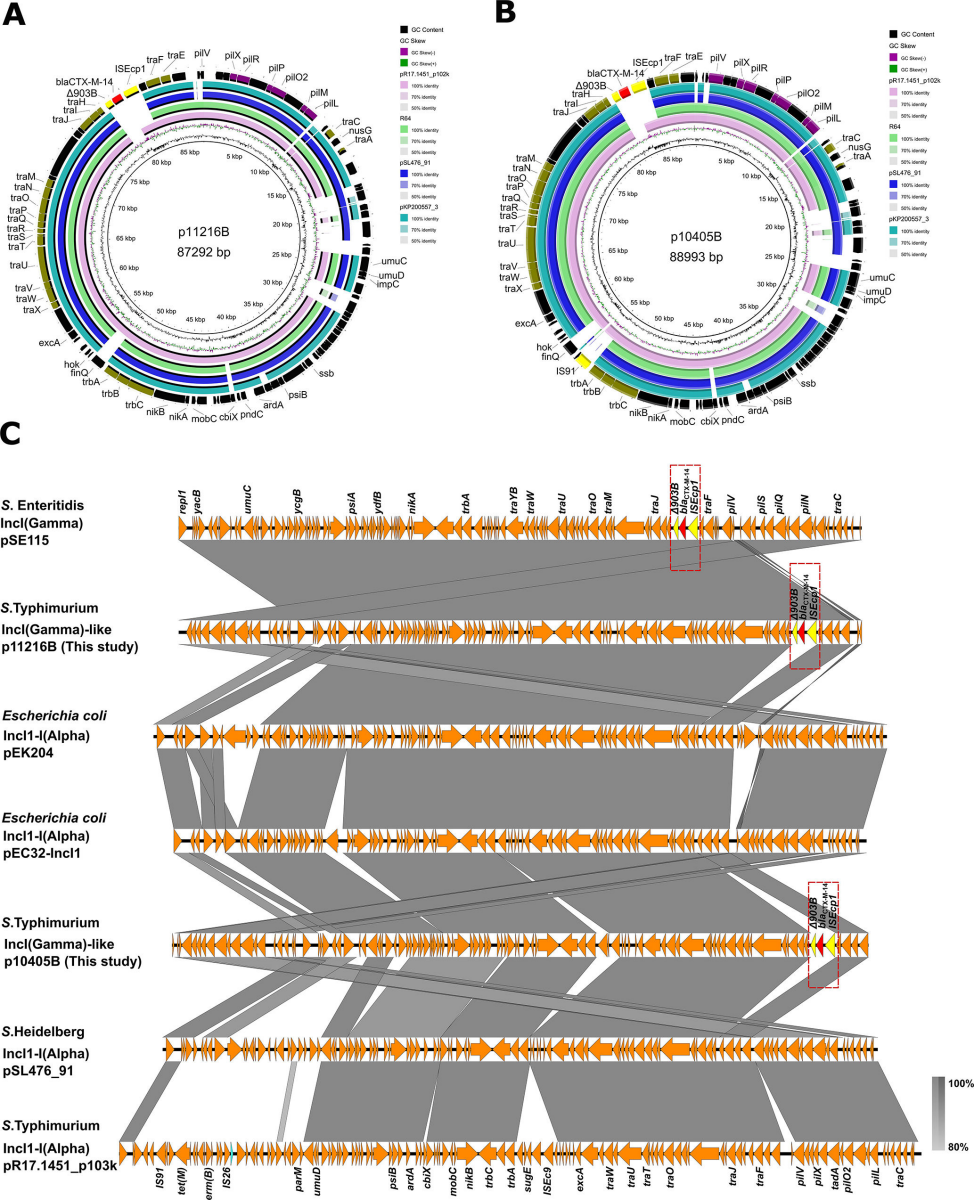
Sequence characterization of p11216B plasmid (**A**) and p10405B plasmid (**B**) in this study. Sequence comparison of IncI (Gamma) plasmids and IncI1-I (Alpha) plasmids (**C**). IncI (Gamma) plasmids included *S*. Enteritidis pSE115 (Accession number KT868530). IncI1-I (Alpha) plasmids included *Escherichia coli* pEK204 (Accession number NC_013120), *Escherichia coli* pEC32-IncI1 (Accession number CP085621), *S*. Heidelberg pSL476_91 (Accession number NC_011081), and S. Typhimurium pR17.1451_p102k (Accession number CP063296). Areas shaded in gray indicate homologies between the corresponding genetic loci.

Two plasmids (p11216B and p10405B) from the SJTUF11216 and SJTUF14504 were further sequenced for analysis (Fig. 4). Plasmids p11216B (Fig. 4A) and p10405B (Fig. 4B) were identified to be 87,292 bp and 88,993 bp in size, respectively. Plasmid p11216B shared 137 of the 141-bp IncI (Gamma) replicon gene (Fig. S3A), and the same result was also found in p10405B (Fig. S3B). Both of these two plasmids belonged to IncI (Gamma)-like types.

It was found that p11216B was highly similar to p10405B (98% coverage; 99.94% identity). In addition, both of these two plasmids showed being highly similar to another IncI1 plasmid pSE115 (Accession number KT868530; 98% coverage; 99.93% identity), which was recovered from a clinical *Salmonella* Enteritidis isolate in China. These plasmids were carried by different hosts, which suggested that the transfer of plasmids might occur through conjugation interspecies. The major difference between p11216B and p10405B was that an IS*91* element was inserted into the backbone between *trb*A and *fin*Q genes (Fig. 4A and B). Gene *bla*_CTX-M-14_ was linked to the transposable elements IS*Ecp1* upstream and ΔIS*903B* downstream (Fig. 4A). Furthermore, this transposon unit was inserted into the conjugational transfer region of the plasmid. The same result was also found in p10405B (Fig. 4B). Mobile genetic elements like insertion sequences could mediate the horizontal transfer of ARGs (32). A previous study demonstrated that IS*Ecp1* could move *bla*_CTX-M-2_ from the chromosome to the plasmid in *Kluyvera ascorbata* through transposition (33). Therefore, we speculated that IS*Ecp1* might play a key role in the horizontal transfer of *bla*_CTX-M_ genes in *S*. Typhimurium.

We further compared these two plasmids in this study with epidemic IncI1-I (Alpha) plasmids (Fig. 4C). It was found that IncI (Gamma) plasmids including p11216B and p10405B shared a highly similar plasmid backbone with IncI1-I (Alpha) plasmids, suggesting that they might be originated from an ancestor. It appeared that the IS*Ecp1-bla*_CTX-M-14_-ΔIS*903B* module was special for IncI (Gamma) plasmids, which was absent for IncI1-I (Alpha) plasmids. The reasons were urgent to be explored in the future.

### Plasmid transfer analysis

Conjugation experiments were carried out to determine the transferability of plasmids p11216B and p10405B with *E. coli* C600 as the recipient. Transconjugants SJTUF11216-TC and SJTUF10405-TC were successfully obtained (Table 4), suggesting that IncI (Gamma)-like plasmids in this study could transfer through conjugation. Conjugation frequencies of SJTUF11216 and SJTUF10405 were 2.55 × 10^−3^ and 9.55 × 10^−4^, respectively, suggesting their strong conjugation transferability. The conjugative regions of self-transmissible mobile genetic elements typically consist of four modules: the origin of transfer site (*oriT*), relaxase gene, gene encoding type IV coupling protein (T4CP), and a gene cluster for the bacterial type IV secretion system (T4SS) (34). The above genetic elements were found in IncI (Gamma)-like plasmids, which may explain their successful transfer by conjugation in *S*. Typhimurium.

**TABLE 4:** Antimicrobial MICs of *S*. Typhimurium SJTUF11216 and SJTUF10405 isolates as well as their transconjugants

Antimicrobial agents	*R* value (μg/mL)	MICs			
		SJTUF11216	SJTUF11216-TC	SJTUF10405	SJTUF10405-TC
*β*-Lactams					
Ampicillin	≥32	≥1,024 (R)	512 (R)	≥1,024 (R)	512 (R)
Ceftriaxone	≥4	16 (R)	16 (R)	16 (R)	16 (R)
Ceftiofur	≥8	64 (R)	16 (R)	64 (R)	32 (R)
Aminoglycosides					
Gentamicin	≥16	1 (S)	0.5 (S)	8 (I)	0.5 (S)
Streptomycin	≥64	32 (I)	≤4 (S)	≥256 (R)	≤4 (S)
Kanamycin	≥64	≥1,024 (R)	≤4 (S)	32 (I)	≤4 (S)
Quinolones					
Nalidixic acid	≥32	≥256 (R)	4 (S)	≥256 (R)	4 (S)
Ciprofloxacin	≥1	1 (R)	0.125 (S)	2 (R)	0.125 (S)
Tetracyclines					
Tetracycline	≥16	128 (R)	1 (S)	128 (R)	1 (S)
Sulphamethoxazole					
Sulfisoxazole	≥512	≥2,048 (R)	128 (S)	≥2,048 (R)	128 (S)
Folate pathway antagonists					
Trimethoprim-sulfamethoxazole	≥4/76	≥32/608 (R)	1/19 (S)	≥32/608 (R)	1/19 (S)
Phenicols					
Chloramphenicol	≥32	256 (R)	4 (S)	256 (R)	4 (S)
Lipopeptides					
Colistin	≥8	≤0.5 (S)	≤0.5 (S)	≤0.5 (S)	≤0.5 (S)
Carbapenems					
Meropenem	≥4	≤0.125 (S)	≤0.125 (S)	≤0.125 (S)	≤0.125 (S)
Imipenem	≥4	≤0.125 (S)	≤0.125 (S)	≤0.125 (S)	≤0.125 (S)

Antimicrobial susceptibility tests showed that SJTUF11216-TC exhibited resistance to ceftriaxone (MIC = 16 µg/mL) as well as other β-lactam antimicrobials such as ampicillin (MIC = 512 µg/mL) and ceftiofur (MIC = 16 µg/mL) (Table 4). SJTUF10405-TC exhibited resistance to ceftriaxone (MIC = 16 µg/mL) as well as other β-lactam antimicrobials such as ampicillin (MIC = 512 µg/mL) and ceftiofur (MIC = 32 µg/mL) (Table 4). Gene *bla*_CTX-M-14_ was identified in transconjugants SJTUF11216-TC and SJTUF10405-TC, suggesting that horizontal transfer of *bla*_CTX-M-14_ on the IncI (Gamma)-like plasmids occurred in the conjugation. The MICs of ciprofloxacin in transconjugants decreased 8–16 times compared with those in the donors, which resulted from the absence of the mutation in *gyr*A in the recipient. These findings suggested that IncI (Gamma)-like plasmids contributed to the horizontal transfer of *bla*_CTX-M-14_ through conjugation.

### Conclusion

The MDR *S*. Typhimurium isolates presented a large-scale prevalence in Shanghai, China, and especially the ceftriaxone and ciprofloxacin co-resistance isolates aroused great concern. The international spread of *S*. Typhimurium isolates was observed, especially the ST34 clone. It was noted that ST34 isolates from Shanghai had fallen into the ST34C2 clade accompanied by ESBL gene *bla*_CTX-M-14_ and a mutation in the *gyr*A 87 site, which might be responsible for the ceftriaxone and ciprofloxacin co-resistance. Our study suggests that necessary strategies such as a national plan and “One Health” theory are warranted to prevent the further dissemination of MDR *S*. Typhimurium, especially the newly emerging ST34C2 clade. We also advocate food enterprises, livestock farms, and hospitals to strictly comply with the national AMR prevention and control document and adopt the “One Health” theory to reduce AMR threat to public health.

## MATERIALS AND METHODS

### Bacterial isolates in this study

A total of 2,211 *Salmonella* isolates from foods, patients, and environments in Shanghai, China, were collected during 2007–2019. The 277 *S*. Typhimurium isolates were then selected in this study, and the rest of serotyped isolates were analyzed in other studies. Food sources included pork, chicken, duck, aquatic products, and others. Human sources included the stool and blood of health checkers, outpatients, and inpatients in hospitals for diarrhea treatment. Environment sources included wastewater and poultry feces. All the above *Salmonella* isolates were identified via API20E test strips (BioMerieux, France) and serotyped via commercial antiserum (Statens Serum Institute, Copenhagen, Denmark) according to the manufacturer’s guidelines.

### Antimicrobial susceptibility testing

Antimicrobial susceptibility testing was performed on *S*. Typhimurium isolates using the agar dilution method provided by the Clinical and Laboratory Standard Institute (35) and the European Committee on Antimicrobial Susceptibility Testing (36). The following antimicrobials were selected: amikacin, ampicillin, ceftiofur, ceftriaxone, nalidixic acid, ciprofloxacin, chloramphenicol, kanamycin, gentamicin, streptomycin, tetracycline, sulfamethoxazole/trimethoprim, sulfisoxazole, meropenem, and imipenem. Bacterial susceptibility to colistin was performed with the broth microdilution method recommended by the European Committee on Antimicrobial Susceptibility Testing. *Escherichia coli* ATCC 25922 and *Enterococcus faecalis* ATCC 29212 were used as quality control strains.

### Whole-genome sequencing

The *S*. Typhimurium isolates were grown in 250 mL lysogeny broth overnight at 37°C, with agitation at 200 rpm. The cell biomass was harvested after 10 min centrifugation at 12,000 × *g*. Genomic DNA was extracted from *S*. Typhimurium isolates using the QIAamp DNA Mini Kit (Qiagen, CA). Genomic DNA was sequenced using a combination of PacBio RS II and Illumina sequencing platforms.

For Illumina sequencing, genomic DNA was used for each strain in sequencing library construction. DNA samples were sheared into 400–500-bp fragments using a Covaris M220 Focused Acoustic Shearer following the manufacturer’s protocol. Illumina sequencing libraries were prepared from the sheared fragments using the NEXTFLEX Rapid DNA-Seq Kit. The prepared libraries then were used for paired-end Illumina sequencing (2 × 150 bp) on Illumina NovaSeq 6000 (Illumina Inc., San Diego, CA, USA). For PacBio sequencing, genomic DNA was fragmented at ~10 kb. DNA fragments were then purified, end-repaired, and ligated with SMRT bell sequencing adapters following the manufacturer’s recommendations (Pacific Biosciences, Menlo Park, CA, USA). Next, the PacBio library was prepared and sequenced on one SMRT cell using standard methods. Sequence data from the PacBio RS II platform were assembled using the Canu software (37). Finally, the consensus genome sequence was determined using the Pilon software (38). The plasmid sequence was extracted from the assembled whole-genome sequence.

Annotation of the genome was performed using RAST (39), BLASTn, and BLASTp programs. The encoding genes in the genome were predicted by Glimmer (40) and GeneMarkS (41). The tRNAs, rRNAs, and repeated sequences in the genome were predicted by tRNAscan-SE v2.0 (http://trna.ucsc.edu/software/), Barrnap (https://github.com/tseemann/barrnap), and Tandem Repeats Finder (http://tandem.bu.edu/trf/trf.html), respectively. The origin of transfers in DNA sequences of bacterial mobile genetic elements was identified using oriTfinder (https://tool-mml.sjtu.edu.cn/oriTfinder/oriTfinder.html).

### AMR determinant analysis

ResFinder 4.1 (https://cge.cbs.dtu.dk/services/ResFinder/) was used to identify ARGs and chromosomal mutations mediating AMR in the genome (42). MLST 2.0 (https://cge.food.dtu.dk/services/MLST/) was used to identify the STs of bacteria (43). PlasmidFinder 2.1 (https://cge.food.dtu.dk/services/PlasmidFinder/) was used to identify replicon types of plasmids (44). ISfinder (https://www-is.biotoul.fr/) was used to analyze the IS and transposons in the genome.

### Phylogenomic analysis

Phylogenomic analysis was performed on genome sequences of *S*. Typhimurium isolates from China (*n* = 135) including 16 genome sequences in this study and other countries (*n* = 266) available from the NCBI public database (by the time 4 June 2021). These 16 isolates for phylogenomic analysis were selected by the number and spectrum of tested antimicrobials. We have added the detailed table (Table S2 ) to explain their AMR characterization. Sequence from *S*. Typhimurium LT2 (Accession number NC_003197) was used as the reference genome. SNPs were extracted using Snippy (https://github.com/tseemann/snippy) to generate core genomic alignment. Gubbins (45) was then used to remove recombination regions. The core SNP alignment was used to generate a maximum likelihood phylogeny using RAxML v8.1.23 (46) with the GTR nucleotide substitution model. Furthermore, 100 random bootstrap replicates were conducted to assess the node support. The phylogenetic tree was visualized together with metadata using Microreact v5.99.0 (47).

A timescale phylogenetic tree of ST34 clone was re-constructed to get the evolutionary history using Beast v2.7.6. The GTR substitution model, random local clock model, and coalescent extended Bayesian skyline model were chosen with BEAUti 2. Markov chain Monte Carlo analyses were run three times with chain lengths of 1.2 × 10^8^ with sampling every 1,000 generations and using a burn-in set at 10%. The effective sample size of all parameters was >200. A timescaled tree was obtained using the maximum sum clade credibility topology with TreeAnnotator v2.6.0.

### Conjugation experiment

Conjugation experiments were performed as described previously (25) with *E.coli* C600 as the recipient. Briefly, *Salmonella* isolates used as the donor were incubated with the recipient overnight, and then, the overnight cultures were mixed and transferred to filter paper on an lysogeny broth (Beijing Landbridge Technology Co. Ltd., China) plate. Transconjugants were selected on MacConkey agar plates supplemented with ceftriaxone (4 µg/mL) and rifampin (200 µg/mL). PCR-based replicon typing using 18 replicon primers was performed on transconjugants as described previously (48). Conjugation frequencies were also calculated as the number of transconjugants per recipient.

### Statistical analysis

The AMR data were analyzed using DPS software 9.5 (Institute of Insect Science, Zhejiang University, Hangzhou, China). Significant (*P* < 0.05) difference in AMR in *S*. Typhimurium isolates from patients and foods during 2007–2019 was determined using the Pearson χ^2^ test at the 5% level (α = 0.05).

The antibiogram length was considered as the number of antimicrobial classes to which an isolate was phenotypically resistant. The MDR was refined as a dependent variable to test whether it differed among sources, years, and locations by utilizing GLMMs as a previous study (49). The “lme4” package of R 4.2.0 (Lucent Technologies, Jasmine Mountain, USA) was used to analyze the antibiogram length, especially MDR data. MDR data were divided into patient and non-patient groups. The isolated years of strains from these groups must be continuous according to the requirements of the GLMMs. Therefore, 185 strains were selected for the analysis of Poisson GLMMs.

## Data Availability

The genome sequence data are available from BioProject ID PRJNA484101. The plasmid sequences of p10405B and p11216B have been deposited in the NCBI database under the accession numbers CP047539 and CP047524, respectively.

## SUPPLEMENTAL MATERIAL

Supplemental figures and tables spectrum.00262-24-s0001.docx Tables S1 and S2; Fig. S1-S3.

ASM does not own the copyrights to Supplemental Material that may be linked to, or accessed through, an article. The authors have granted ASM a non-exclusive, world-wide license to publish the Supplemental Material files. Please contact the corresponding author directly for reuse.
