# Supplementary material for: Dynamic antimicrobial resistance and phylogenomic structure of Salmonella Typhimurium from 2007 to 2019 in Shanghai, China
Source: Microbiol Spectr. 2024 Jun 21;12(8):e00262-24. doi: 10.1128/spectrum.00262-24 (PMC11302141; doi:10.1128/spectrum.00262-24)
Supplement: Supplemental figures and tables — Tables S1 and S2; Fig. S1-S3. [file spectrum.00262-24-s0001.docx]

**Table S1** Antimicrobials resistance of 146 *S*. Typhimurium isolates from foods in Shanghai, China

| Antimicrobials | Resistant isolates (No. / ratio %) | | | Total (n=146) |
| --- | --- | --- | --- | --- |
|  | Meats (n=96) | Aquatic products (n=22) | Others (n=28) |  |
| ***β*-Lactams** |  |  |  |  |
| Ampicillin | 61/63.5 | 13/59.1 | 11/39.3 | 85/58.2 |
| Ceftriaxone | 1/1.0 | 0/0.0 | 0/0.0 | 1/0.7 |
| Ceftiofur | 1/1.0 | 0/0.0 | 0/0.0 | 1/0.7 |
| **Aminoglycosides** |  |  |  |  |
| Amikacin | 0/0.0 | 0/0.0 | 0/0.0 | 0/0.0 |
| Gentamicin | 42/43.8 | 9/40.9 | 9/32.1 | 60/41.1 |
| Streptomycin | 47/49.0 | 12/54.5 | 10/35.7 | 69/47.3 |
| Kanamycin | 45/46.9 | 8/36.4 | 12/42.9 | 65/44.5 |
| **Quinolones** |  |  |  |  |
| Nalidixic acid | 62/64.6 | 14/63.6 | 20/71.4 | 96/65.8 |
| Ciprofloxacin | 6/6.3 | 3/13.6 | 1/3.6 | 10/6.8 |
| **Tetracyclines** |  |  |  |  |
| Tetracycline | 55/57.3 | 12/54.5 | 14/50.0 | 81/55.5 |
| **Sulphamethoxazole** |  |  |  |  |
| Sulfisoxazole | 93/96.9 | 22/100.0 | 27/96.4 | 142/97.3 |
| **Folate pathway antagonists** |  |  |  |  |
| Trimethoprim-sulfamethoxazole | 56/58.3 | 11/50.0 | 11/39.3 | 78/53.4 |
| **Phenicols** |  |  |  |  |
| Chloramphenicol | 39/40.6 | 11/50.0 | 11/39.3 | 61/41.8 |
| **Lipopeptides** |  |  |  |  |
| Colistin | 0/0.0 | 0/0.0 | 0/0.0 | 0/0.0 |
| **Carbapenems** |  |  |  |  |
| Meropenem | 0/0.0 | 0/0.0 | 0/0.0 | 0/0.0 |
| Imipenem | 0/0.0 | 0/0.0 | 0/0.0 | 0/0.0 |
| ≥3 (MDR) | 76/79.2 | 14/63.6 | 17/60.7 | 107/73.3 |
| ≥5 | 56/58.3 | 13/59.1 | 9/32.1 | 78/53.4 |
| ≥7 | 8/8.3 | 3/13.6 | 1/3.6 | 12/8.2 |

**Table S2** Antimicrobials resistance phenotype of selected *S*. Typhimurium isolates for genome sequencing

| Strains | Group | MIC (µg/mL) | | | | | | | | | | | | | | | |
| --- | --- | --- | --- | --- | --- | --- | --- | --- | --- | --- | --- | --- | --- | --- | --- | --- | --- |
|  |  | CRO | TIO | CIP | NAL | GEN | KAN | STR | TET | CHL | AMP | FIS | SXT | COL | MEP | IMP |  |
| SJTUF10484 | 1 | - | - | - | - | - | - | - | R | - | R | R | R | - | - | - |  |
| SJTUF10231 |  | - | - | - | - | - | - | R | R | - | R | R | R | - | - | - |  |
| SJTUF10359 |  | - | - | - | - | R | - | - | - | R | R | R | R | - | - | - |  |
| SJTUF10057 | 2 | - | - | - | - | R | - | R | R | R | R | R | R | - | - | - |  |
| SJTUF10112 |  | - | - | - | - | R | R | R | R | R | R | R | R | - | - | - |  |
| SJTUF10452 |  | - | - | - | - | R | R | - | - | R | R | R | R | - | - | - |  |
| SJTUF10330 |  | - | - | - | R | R | - | R | R | R | R | R | R | - | - | - |  |
| SJTUF10640 |  | - | - | - | R | R | R | - | - | R | R | R | R | - | - | - |  |
| SJTUF10236 | 3 | - | - | R | R | - | R | - | R | R | R | R | R | - | - | - |  |
| SJTUF10250 |  | - | - | R | R | - | - | R | R | R | R | R | R | - | - | - |  |
| SJTUF10169 |  | - | - | R | R | R | - | - | R | R | R | R | R | - | - | - |  |
| SJTUF10648 |  | - | - | R | R | R | R | R | R | R | R | R | R | - | - | - |  |
| SJTUF11077 |  | - | - | R | R | R | R | R | R | R | R | R | R | - | - | - |  |
| SJTUF11216 | 4 | R | R | R | R | - | R | - | R | R | R | R | R | - | - | - |  |
| SJTUF10405 |  | R | R | R | R | - | - | R | R | R | R | R | R | - | - | - |  |
| SJTUF10855 |  | R | R | R | R | - | - | - | R | R | R | R | R | - | - | - |  |

Note: The *Salmonella* Typhimurium for phylogenomic analysis were selected by the number and spectrum of tested antimicrobials. These 16 strains examined in this study were roughly divided into 4 groups. The Group 1 was resistant to 4-5 commonly used antimicrobials, such as ampicillin, sulfamethoxazole/trimethoprim, sulfisoxazole, streptomycin, tetracycline, chloramphenicol and gentamicin. The Group 2 exhibited variable resistance to 6–8 commonly used antimicrobials, such as ampicillin, sulfamethoxazole/trimethoprim, sulfisoxazole, streptomycin, tetracycline, chloramphenicol, kanamycin, nalidixic acid and gentamicin. Compared to those in Group 2, the Group 3 showed resistance to 8–10 commonly used antimicrobials including additional new resistance to ciprofloxacin. Compared to those in Group 3, the Group 4 showed resistance to 8–10 commonly used antimicrobials including additional new resistance to ciprofloxacin, ceftriaxone and ceftiofur. “-” stands for the antimicrobial susceptibility.


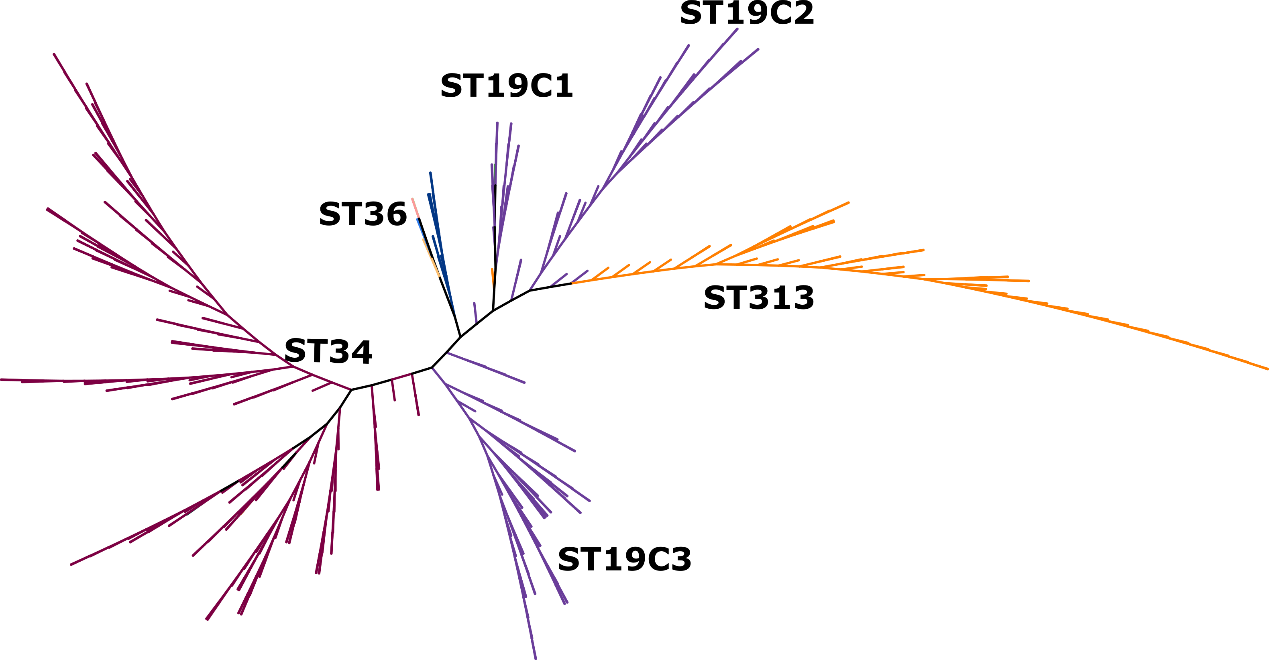


**Fig. S1**. No-root phylogenetic tree of 401 *S*. Typhimurium genomes.


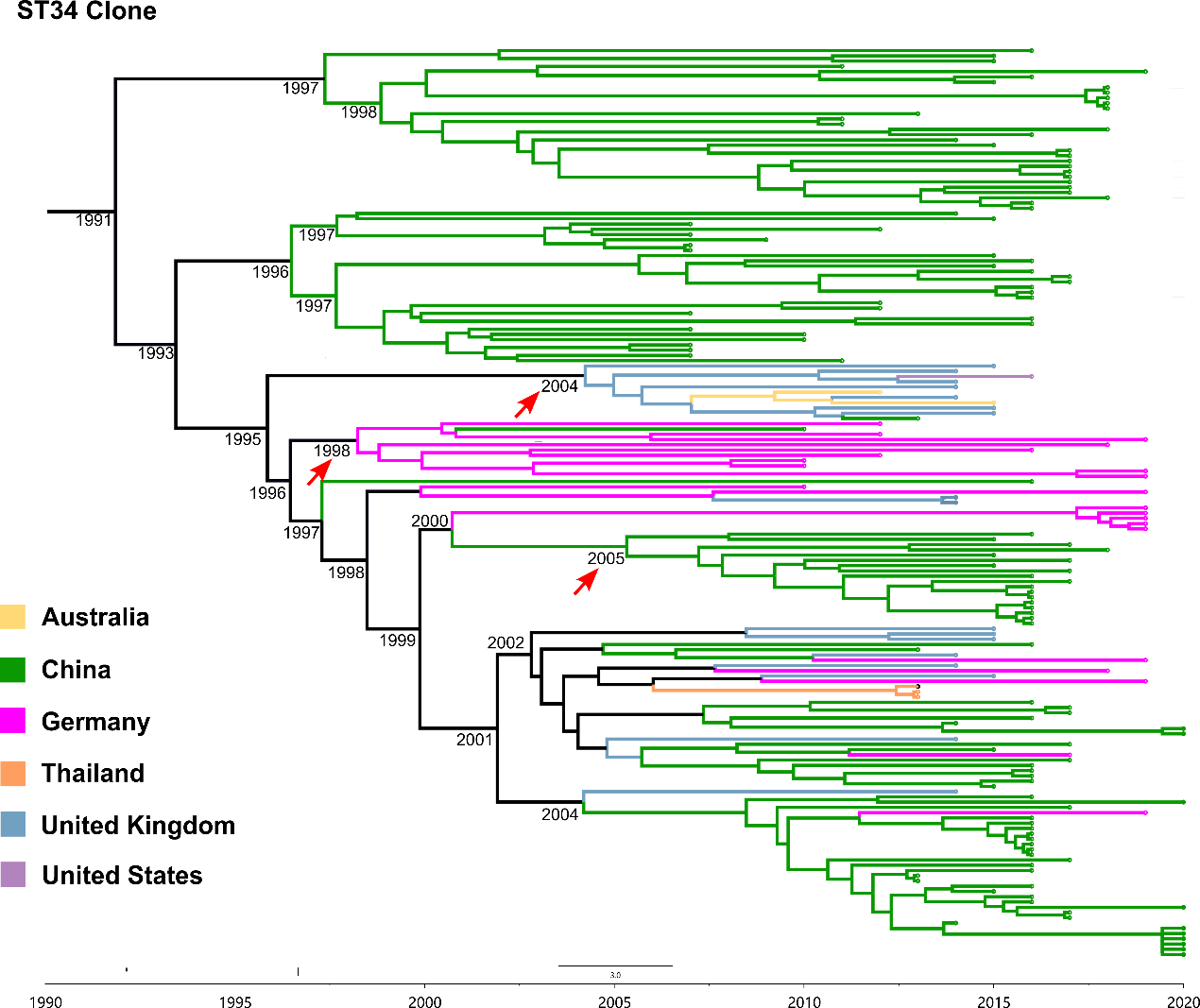


**Fig. S2**. A reconstructed time-scale phylogenomic tree of *S*. Typhimurium ST34 clone in Fig. 2C using Beast v2.7.6. Arrows point to the main evolutionary location jumps.

**A**


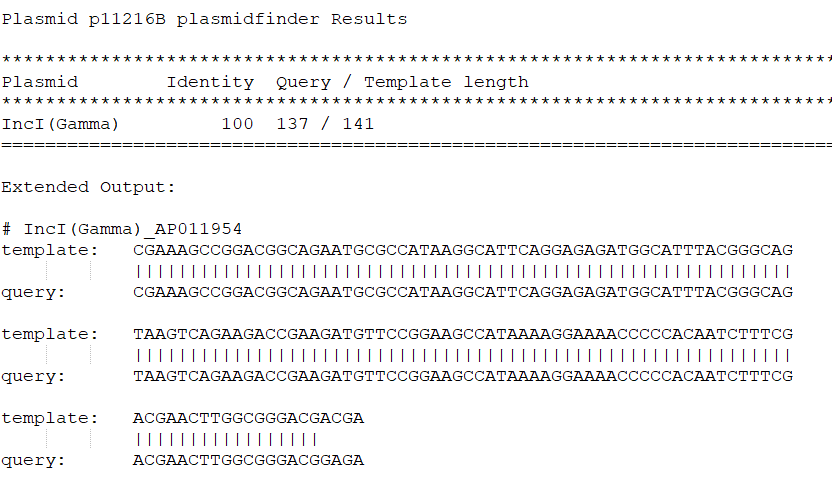


**B**


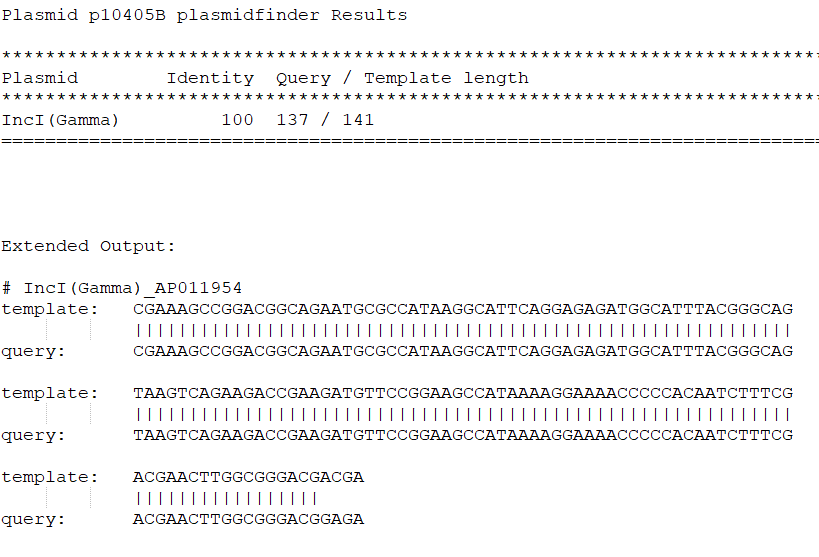


**Fig. S3**. The plasmidfinder results of plasmid p11216B (A) and p10405B (B)
